# Axial Elongation During Short-Term Accommodation in Myopic and Nonmyopic Children

**DOI:** 10.1167/iovs.63.3.12

**Published:** 2022-03-11

**Authors:** Rohan P. J. Hughes, Scott A. Read, Michael J. Collins, Stephen J. Vincent

**Affiliations:** 1 Queensland University of Technology, Contact Lens and Visual Optics Laboratory, Centre for Vision and Eye Research, School of Optometry and Vision Science, Brisbane, Queensland, Australia

**Keywords:** accommodation, ocular biometry, axial length, near work, myopia

## Abstract

**Purpose:**

Axial length increases during accommodation in adults and children; however, refractive error group differences are conflicting and have not been explored in pediatric populations. This study aimed to evaluate differences in accommodation-induced axial elongation between myopic and nonmyopic children.

**Methods:**

A range of ocular biometric measurements were captured during brief accommodation tasks (0, 3, 6, and 9 D) using a Badal optometer mounted to a noncontact optical biometer (Zeiss IOLMaster 700). Reliable measurements were captured for 15 myopic and 15 age- and sex-matched nonmyopic children. The average central corneal thickness (CCT), anterior chamber depth (ACD), crystalline lens thickness (LT), anterior segment length (ASL), vitreous chamber depth (VCD), and axial length (AL) were determined for each accommodation stimulus. Raw measurements of AL and VCD were corrected for the estimated error associated with LT increases during accommodation.

**Results:**

All biometric parameters, except CCT, changed significantly during accommodation (all *P* < 0.001). Myopic children exhibited significantly greater accommodation-induced axial elongation than nonmyopic children (*P* = 0.002) at the 3, 6, and 9 D accommodation stimuli, with a mean difference of 7, 10, and 16 µm, respectively (all pairwise comparisons, *P* ≤ 0.03). The changes in all other biometric parameters were not different between the refractive error groups (*P* ≥ 0.23).

**Conclusions:**

Accommodation-induced axial elongation was greater in myopic than nonmyopic children. This finding could support a potential mechanism linking near work, axial elongation, and myopia development in children or may reflect greater susceptibility to accommodation-induced axial elongation in children with established myopia.

Structural components of the accommodation apparatus differ between myopes and nonmyopes, with a thinner crystalline lens^1^ and thicker ciliary muscle^2^ and ciliary body^3^^,^^4^ typically observed in myopes. Several functional aspects of accommodation also differ between myopes and nonmyopes. For the same accommodation stimulus, myopic adults^5^^–^^7^ and children^8^^,^^9^ often exhibit a greater lag of accommodation compared to age-matched emmetropes, which may be due to structural or functional differences in the ciliary muscle and body.^4^^,^^10^ Additionally, Mutti et al.^11^ suggested that children who develop myopia may require greater accommodative effort because of accommodative-vergence dysfunction, as evidenced by an increase in the AC/A ratio observed during the four years before myopia development,^11^ which remained elevated compared to emmetropic children.^11^^–^^13^ These findings suggest differences in accommodative function between myopes and emmetropes. Given these structural and functional differences during accommodation associated with refractive error, it is possible that the magnitude of the ocular biometric changes during accommodation also differs between myopic and nonmyopic children.

In young adults, several studies have shown that similar amounts of anterior chamber shallowing^14^^–^^16^ and crystalline lens thickening^14^^,^^16^ occur during accommodation in myopes and emmetropes. However, differences in the magnitude of axial elongation during accommodation have been reported between refractive error groups of young adults, although the findings are conflicting. Drexler et al.^14^ found that the axial length (AL) of emmetropes increased significantly more than myopes during accommodation, although the AL measurements were captured with a target placed at their near point of accommodation (i.e., their maximum amplitude of accommodation), resulting in emmetropes accommodating ∼1 D more than myopes on average. Mallen et al.^15^ reported the opposite finding, with significantly greater axial elongation in myopes compared to emmetropes for a 6 D accommodation stimulus, whereas Read et al.^16^ found no significant difference in axial elongation between emmetropic and myopic young adults up to a 6 D accommodation stimulus. Similarly, Aldossari et al.^17^ reported no significant difference in accommodation-induced axial elongation between low myopes and emmetropes for a 6 D stimulus, but they observed a moderate, statistically significant correlation between increasing levels of myopia and greater accommodation-induced axial elongation, which suggests that individuals with greater levels of myopia may be more susceptible to accommodation-induced axial elongation.

These prior studies of adults with established myopia suggest that there may be a difference in the magnitude of accommodation-induced axial elongation between myopes and nonmyopes, particularly for those with higher levels of myopia. We have previously reported accommodation-induced axial elongation in nonmyopic children,^18^ with the magnitude of AL changes similar to those of emmetropic adults for accommodation stimuli up to 6 D^14^^–^^16^^,^^19^^,^^20^; however, refractive error group differences in accommodation-induced axial elongation have not previously been examined in children. Therefore, the primary aim of this study was to examine changes in AL and a range of ocular biometric parameters during accommodation between a group of school-aged myopic children and a group of age- and sex-matched nonmyopic children. This study also explored the association between changes in the anterior segment and changes in AL during accommodation to evaluate the contributions of individual ocular components to accommodation-induced axial elongation and any possible refractive error group differences. Finally, the study sought to establish whether, in children as reported in adults, longer eyes show greater accommodation-induced axial elongation.

## Methods

Eighteen myopic children (11 males and seven females) were recruited, with a mean age (± SD) of 10.1 ± 1.4 years (range, 7.3–12.7 years) and noncycloplegic spherical equivalent refraction (SER) of −2.08 ± 0.92 D (range, −0.75 to −3.50 D). All were habitually corrected with single-vision distance spectacles, and none had previously used any optical (spectacle or contact lens) or pharmacological myopia control interventions. The myopic children were all in good general and ocular health, had no significant binocular vision anomalies, and exhibited ≥ 9 D amplitude of accommodation as determined with the push-up method using an N6 target.

Eighteen nonmyopic children (11 males and seven females) from a previous study^18^ were age- and sex-matched to the myopic children, selected first for sex and then age. A minimum sample size of 18 (nine participants in each refractive error group) was determined using G*Power^21^ to achieve 80% statistical power with an alpha-error level of 0.05 to detect an interaction between accommodation stimulus and refractive error group using preliminary data from two myopes and two nonmyopes. Ethics approval was granted by the Queensland University of Technology Human Research Ethics Committee, all participants gave informed assent, and their parents or guardians provided informed consent before study participation.

**Figure 1.**
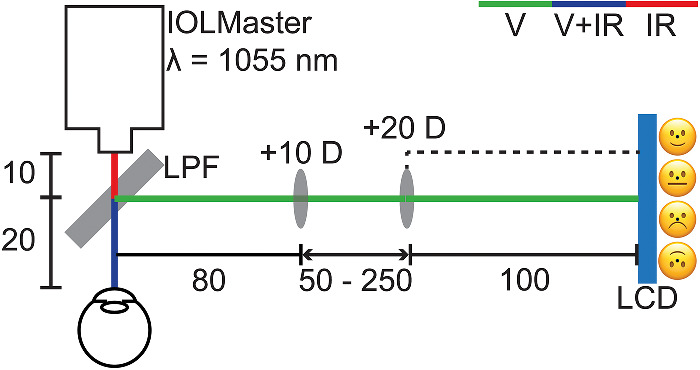
Schematic diagram of the Badal optometer attached to the Zeiss IOLMaster 700 optical biometer. The four emoticon fixation targets are also displayed. All distances are expressed in millimeters. LPF, longpass dichroic filter; LCD, liquid crystal display; V, “visible” radiation (400–650 nm); IR, “infrared” radiation (>650 nm). Figure reproduced from Hughes et al.^18^ with permission from John Wiley and Sons.

After a screening assessment to determine eligibility, each participant underwent a washout period of at least five minutes during which time they watched an age-appropriate video on a screen at 3 m with habitual correction, to minimize the influence of prior visual activities. Ocular biometry of the left eye was measured using an optical biometer (IOLMaster 700; Carl Zeiss Meditec AG, Jena, Germany), to which a Badal optometer was attached to present four accommodation stimuli (0, 3, 6, and 9 D) (Fig. 1). The optometer consisted of a longpass dichroic filter (LPF) placed 20 mm from the eye, angled at 45°, a +10 D lens positioned 80 mm from the LPF (an optical distance of 100 mm from the participant's eye), and an auxiliary +20 D lens fixed at 100 mm from a liquid crystal display. The separation between the lenses could be varied between 50 and 250 mm to present different accommodation stimuli. The LPF reflected wavelengths of 400–650 nm and transmitted wavelengths >650 nm, which allowed optical biometry measurements to be captured while the participant fixated a target (one of four different emoticons) displayed on the liquid crystal display imaged through the Badal optometer.

Prior to measurement capture, the Badal optometer was calibrated to present a 0 D accommodation stimulus (based on the non-cycloplegic SER) and the IOLMaster 700 was aligned to the correct height and distance from the participant's eye. The participant provided verbal directions to align the instrument fixation target (red light) to the center of the emoticon target prior to measurements being captured. The Badal optometer was adjusted to blur the emoticon target by 2-3 D and the participant was asked to indicate when it became clear as it was slowly returned to the 0 D accommodation stimulus position, to ensure accommodation was relaxed prior to measurements.

**Figure 2.**
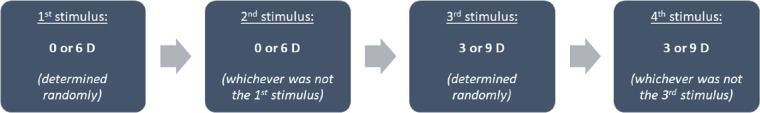
Flow chart to illustrate the semirandomized presentation order for the 0, 3, 6, and 9 D accommodation stimuli.

Several strategies were incorporated into the procedures to minimize potential data loss because of a loss of the child's engagement, fixation, or fatigue. Accommodation stimuli were presented in a semirandomized order to reduce the possible systematic influence of ascending-order stimuli presentation while minimizing potential data loss from a single measurement capture due to task disengagement. The 0 or 6 D stimulus was presented initially (randomly determined), followed by whichever was not presented first. The third accommodation stimulus presented was either the 3 or 9 D stimulus (randomly determined), and the remaining stimulus was presented last (Fig. 2).

Additionally, four different emoticon targets (Fig. 1) were used as the stimulus to accommodation to maintain the interest and fixation of the children during measurements. The targets and their smallest spatial detail subtended 206 minutes of arc and seven minutes of arc at the ocular plane, respectively. The presentation order of the four targets were fully randomized to minimize any potential systematic effects and make the task more engaging by asking the children to describe the expression of the emoticon. Participants were also allowed frequent short breaks from accommodating and positioning in the chin and head rest between the presentation of each stimulus.

One measurement capture was obtained for each participant at each accommodation stimulus. The children were also encouraged to actively maintain fixation and clarity of the targets during measurements, which required 30 to 60 seconds of engagement for each measurement. If they reported that the target was blurred for a particular accommodation stimulus, their data were recaptured for that stimulus and excluded if they were unable to maintain clarity of the target.

Each measurement capture consisted of six meridional B-scans, from which on-axis measurements of the central corneal thickness (CCT), anterior chamber depth (ACD), crystalline lens thickness (LT), anterior segment length (ASL), vitreous chamber depth (VCD), and AL were determined. Individual B-scans were excluded from analyses if the alignment or image quality was poor, with an average of 5.9 ± 0.5 B-scans for each measurement capture included in the analysis. From the remaining B-scans, the mean CCT, ACD, LT, ASL, VCD, and AL were determined for each participant for each accommodation stimulus. Because of the overestimation in VCD and AL measurements captured using optical biometers resulting from the LT increase,^18^^,^^22^ these measurements were also corrected (cVCD and cAL) to account for the estimated errors.

An active accommodation response was expected to produce an LT increase and an ACD decrease; therefore, the change in LT and ACD from the 0 D stimulus was calculated for each participant at each accommodation stimulus, and data were only included if the change in ACD was < 0 µm and LT was > 0 µm for any accommodation stimulus. Where the change in ACD or LT did not meet these criteria, data for all biometric parameters for that participant for the particular accommodation stimulus were excluded from further analysis. This resulted in the exclusion of data for two nonmyopes for the 3 D stimulus and one nonmyope for the 9 D stimulus, who exhibited an increase in ACD (i.e., > 0 µm) or decrease in LT (i.e. < 0 µm) of at least 5 µm for an accommodation stimulus. For data included in the analyses, the minimum change in ACD and LT for the 3 D accommodation stimulus was −7 µm and 21 µm, respectively, with all changes for the 6 and 9 D stimuli greater in magnitude.

A series of linear mixed model (LMM) analyses were carried out for each biometric parameter (CCT, ACD, LT, ASL, cVCD, and cAL) with fixed factors of accommodation stimulus (0, 3, 6, and 9 D) and refractive error group (myopic and nonmyopic) and their interaction. The individual participant's intercepts were included as random effects in the model, and a “variance components” matrix covariance structure was used for the random effects and repeated measures. Missing data were accounted for using a “restricted maximum likelihood” strategy. Post-hoc pairwise comparisons with a Sidak adjustment were used to compare between accommodation stimuli. Where a significant interaction between refractive error group and accommodation stimulus was found for any of the biometric parameters, data were transformed to the change in each parameter from 0 D at each accommodation stimulus, and additional LMM analyses were undertaken using the same fixed factors and strategies. Post-hoc pairwise comparisons were also conducted with a Sidak correction to evaluate the difference between the refractive groups at each accommodation stimulus.

Additional LMM analyses using the same strategies were undertaken to examine the secondary study aims. First, the association between accommodation-induced anterior segment changes and axial elongation between the myopic and nonmyopic children was examined by exploring the interaction between the changes in ACD, LT, and ASL individually, the change in cAL, and refractive error group. Second, the interaction between accommodation stimulus and baseline AL (with all variables also included in the analyses individually) was evaluated to determine if the baseline AL (at the 0 D stimulus) was associated with the magnitude of the axial elongation at each accommodation stimulus. Pearson correlations were also conducted to analyze the strength of these associations.

## Results

### Participant Characteristics

Three of the myopic participants (two females and one male) were excluded from the analysis because of an inability to maintain fixation or clarity of the stimulus target or a lack of accommodation response based on no observable changes in ACD and LT, with the three age- and sex-matched nonmyopic children also excluded, leaving 15 participants for each refractive error group included in the analyses. The age of the remaining myopic (10.2 ± 1.5 years) and nonmyopic participants (10.1 ± 1.2 years) was similar (independent samples *t*-test, *P* = 0.83).

For the 3, 6, and 9 D accommodation stimuli, data for some participants were unable to be reliably captured and some were deemed to not be actively accommodating based on the anterior segment changes; therefore, the sample was reduced at the higher accommodation stimuli for both refractive error groups. LMM analyses confirmed that age did not differ between the nonmyopes and myopes on average across all accommodation stimuli (*P* = 0.52), and there were no differences between the groups for each accommodation stimulus (all pairwise comparisons *P* ≥ 0.65). The age and SER of the included participants in the myopic and nonmyopic groups at each accommodation stimulus are presented in Table 1.

**Table 1.** Sample Size (N), Mean (± SD) Age, and SER for the Included Myopic and Nonmyopic Participants at Each Accommodation Stimulus

		Accommodation Stimulus (D)			
	Refractive Error Group	0	3	6	9
N	Nonmyopes	15	14	15	11
	Myopes	15	15	15	10
Age (years)	Nonmyopes	10.1 ± 1.2	10.0 ± 1.2	10.1 ± 1.2	10.1 ± 1.4
	Myopes	10.2 ± 1.5	10.2 ± 1.5	10.2 ± 1.5	10.3 ± 1.5
SER (D)	Non-myopes	0.66 ± 0.25	0.67 ± 0.26	0.66 ± 0.25	0.67 ± 0.18
	Myopes	−2.05 ± 0.96	−2.05 ± 0.96	−2.05 ± 0.96	−1.93 ± 1.02

LMM analyses revealed no significant difference in the mean age or SER of the myopic and nonmyopic groups associated with accommodation stimulus (*P* > 0.05).

Table 2 presents the mean (± SEM) measurement of each biometric parameter for relaxed accommodation (0 D stimulus) and the mean change in each parameter at each accommodation stimulus for all participants, and for the nonmyopic and myopic groups.

**Table 2.** Mean (± SEM) measurement of CCT, ACD, LT, ASL, cVCD, and cAL for Relaxed Accommodation (0 D Stimulus), and the Mean Change (± SEM) in These Parameters at Each Accommodation Stimulus for All Participants, and the Nonmyopic and Myopic Groups Separately

			Mean Change From the 0 D Accommodation Stimulus (µm)			*P* Value		
Biometric Parameter	Refractive Error Group	0 D (mm)	3 D	6 D	9 D	Accommodation Stimulus	Refractive Group	Accommodation Stimulus By Refractive Group
CCT						0.20	0.70	0.25
	Nonmyopes	0.539 ± 0.011	−1 ± 1	−1 ± 1	0 ± 1			
	Myopes	0.543 ± 0.005	0 ± 2	0 ± 1	−1 ± 1			
	All	0.541 ± 0.006	−1 ± 0	0 ± 0	−1 ± 1			
ACD						**< 0.001**	**< 0.001**	0.23
	Nonmyopes	3.06 ± 0.07	−100 ± 11	−201 ± 19	−320 ± 26			
	Myopes	3.30 ± 0.08	−92 ± 14	−238 ± 13	−313 ± 32			
	All	3.18 ± 0.06	−96 ± 8	−220 ± 12	−317 ± 16			
LT						**< 0.001**	0.22	0.27
	Nonmyopes	3.44 ± 0.05	142 ± 15	278 ± 28	434 ± 33			
	Myopes	3.35 ± 0.04	115 ± 15	326 ± 20	443 ± 45			
	All	3.40 ± 0.03	128 ± 10	302 ± 17	438 ± 22			
ASL						**< 0.001**	0.12	0.23
	Nonmyopes	7.05 ± 0.06	41 ± 7	76 ± 12	114 ± 14			
	Myopes	7.19 ± 0.07	23 ± 4	88 ± 10	129 ± 14			
	All	7.12 ± 0.05	32 ± 4	82 ± 8	121 ± 8			
cVCD						**< 0.001**	**< 0.001**	0.33
	Nonmyopes	16.03 ± 0.17	−33 ± 7	−67 ± 11	−95 ± 15			
	Myopes	17.39 ± 0.19	−9 ± 4	−69 ± 10	−95 ± 13			
	All	16.71 ± 0.17	−21 ± 4	−68 ± 7	−95 ± 8			
cAL						**< 0.001**	**< 0.001**	**0.002**
	Nonmyopes	23.07 ± 0.15	8 ± 2	9 ± 2	18 ± 3			
	Myopes	24.58 ± 0.17	14 ± 3	19 ± 2	34 ± 4			
	All	23.83 ± 0.17	11 ± 2	14 ± 2	26 ± 2			

Note the different units used for the measurement of each parameter for the 0 D stimulus (mm) and the mean changes during accommodation (µm). *P* values are the result of the LMM analyses for the fixed factors of accommodation stimulus and refractive error group, and the interaction between these factors. Significant *P* values are in bold font.

### Change in Ocular Biometry During Accommodation

Considering all participants from both refractive error groups together, cAL increased significantly with accommodation (*P* < 0.001). The mean (± SEM) cAL increase was 11 ± 2 µm at 3 D, 14 ± 2 µm at 6 D, and 26 ± 2 µm at 9 D, with all pairwise comparisons highly significant (all *P* < 0.001), except the difference between the 3 and 6 D stimuli (*P* = 0.48). Each anterior eye parameter also changed significantly with accommodation (all *P* < 0.001), except CCT (*P* = 0.20). The greatest change occurred for LT, which increased by 128 ± 10, 302 ± 17, and 438 ± 22 µm at the 3, 6, and 9 D accommodation stimuli, respectively (all pairwise comparisons *P* < 0.001). The posterior surface of the crystalline lens moved posteriorly, indicated by the significant change in ASL, which increased by 32 ± 4 µm at 3 D, 82 ± 8 µm at 6 D and 121 ± 8 µm at 9 D (all pairwise comparisons *P* < 0.001). ACD decreased significantly with accommodation by −96 ± 8 µm at 3 D, −220 ± 12 µm at 6 D, and −317 ± 16 µm at 9 D (all pairwise comparisons *P* < 0.001). The cVCD also decreased significantly during accommodation with a reduction of −21 ± 4, −68 ± 7, and −95 ± 8 µm at the 3, 6, and 9 D accommodation stimuli, respectively. All pairwise comparisons were significant (all *P* ≤ 0.05) except for the difference between cVCD at 0 and 3 D (*P* = 0.16).

### Comparison Between Myopic and Nonmyopic Children

**Figure 3.**
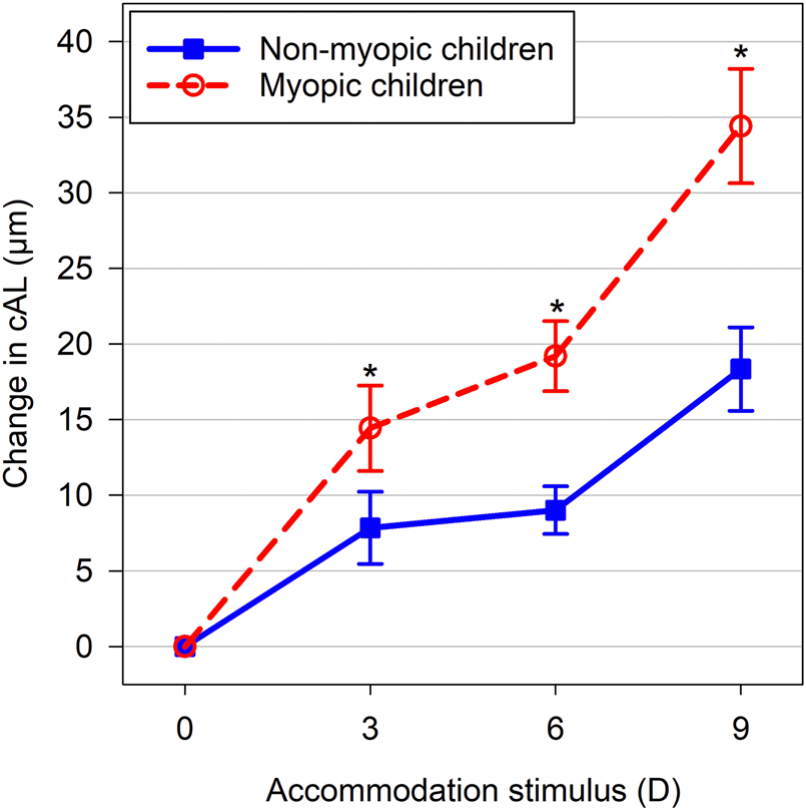
Mean change in cAL during accommodation for the nonmyopic (*solid line/filled squares*) and myopic children (*dashed line/open circles*). The *error bars* represent the SEM. *Asterisks* indicate significant pairwise differences in the mean change between refractive error groups (all *p* ≤ 0.03).

**Figure 4.**
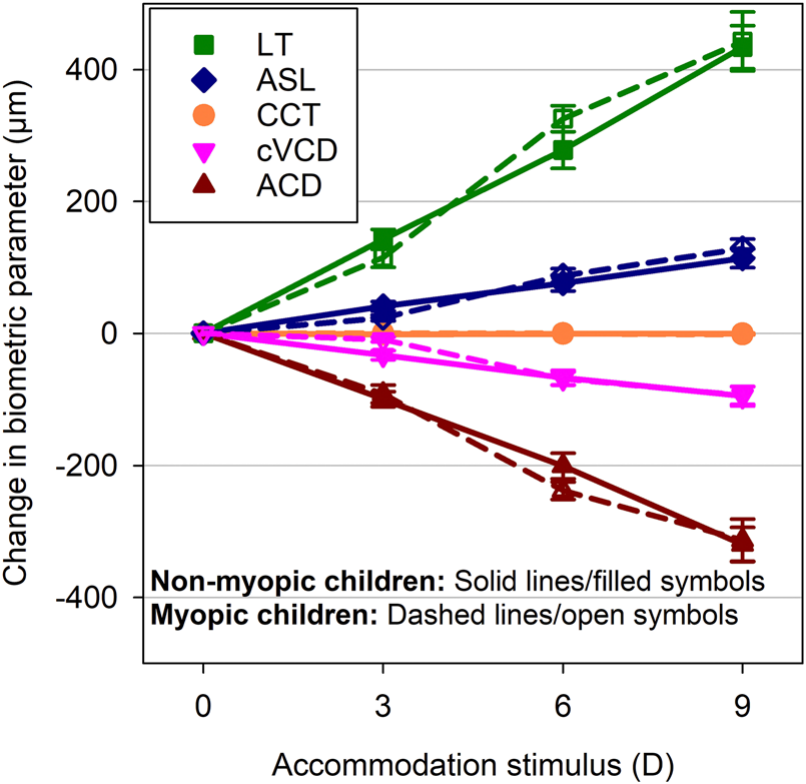
The mean change in CCT, ACD, LT, ASL, and cVCD during accommodation for the nonmyopic (*solid lines/filled symbols*) and myopic children (*dashed lines/open symbols*). The *error bars* represent the SEM.

On average, the myopic children exhibited significantly greater ACD, cVCD, and cAL than the nonmyopic children (all *P* < 0.001), but both refractive error groups had a similar CCT, LT, and ASL (all *P* ≥ 0.12). A significant refractive error group by accommodation stimulus interaction was observed for cAL (*P* = 0.002). The myopic children exhibited a greater increase in cAL with accommodation than the nonmyopes (*P* = 0.001), with a statistically significant difference between the groups observed at the 3, 6, and 9 D stimuli (all *P* ≤ 0.03) and the magnitude of the difference between the refractive error groups increasing with greater accommodation (Fig. 3). There was no significant interaction between accommodation stimulus and refractive error group for all other biometric parameters (CCT, ACD, LT, ASL, and cVCD) (all *P* ≥ 0.23) (Fig. 4).

### Association Between Accommodation-Induced Axial Length and Anterior Segment Changes in Myopic and Nonmyopic Children

**Figure 5.**
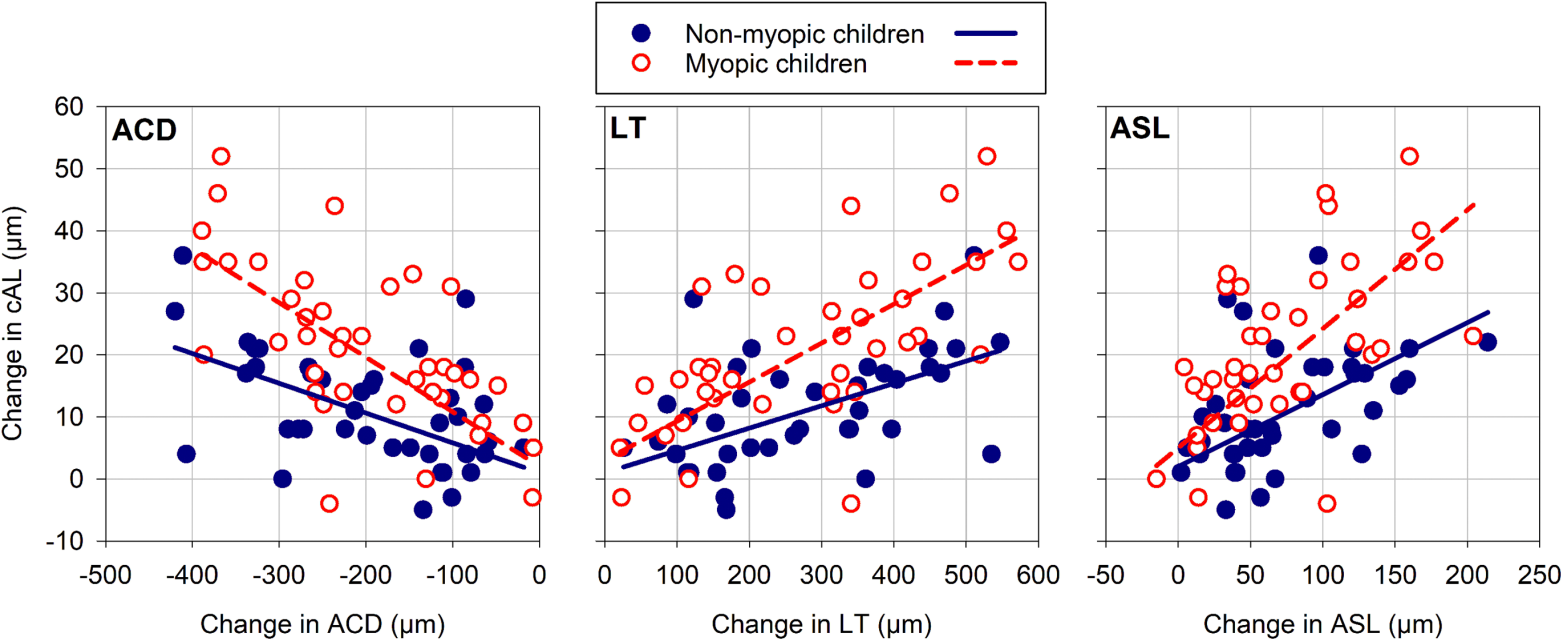
Scatterplots demonstrating the associations between the change in cAL and the change in ACD, LT, and ASL for the nonmyopic (*filled circles*) and myopic children (*open circles*). The *solid* (nonmyopic children) and *dashed lines* (myopic children) represent the linear regression fits as calculated from the estimate of fixed effects in the LMM analyses.

There was a significant interaction between refractive error group and the change in cAL during accommodation and the accommodation-induced changes in ACD (*P* < 0.001), LT (*P* < 0.001), and ASL (*P* = 0.007). Significant slopes were observed for ACD, LT, and ASL for both refractive error groups (all *P* < 0.001). The myopic children exhibited the steepest estimated slopes (± SE of the estimated slope) of −0.088 ± 0.008 for ACD, 0.063 ± 0.005 for LT, and 0.192 ± 0.019 for ASL, compared to the slopes for the nonmyopic children of −0.048 ± 0.008 for ACD, 0.036 ± 0.006 for LT, and 0.116 ± 0.021 for ASL (Table 3). Based on these estimated slopes, the accommodation-induced axial elongation for the myopic children was ∼83%, ∼75%, and ∼66% greater than the nonmyopic children for the same amount of change in ACD, LT, and ASL, respectively (Fig. 5).

**Table 3.** Estimated Linear Regression Slopes and SE, and Pearson Correlation Coefficients and *P* Values for the Association Between the Change in cAL and the Changes in ACD, LT, and ASL for the Nonmyopic and Myopic Children^*^

		Estimate of Fixed Effects (LMM)		Pearson Correlations	
Anterior Segment Parameter	Refractive Error Group	Slope	SE	*r*	*P* Value
ACD					
	Nonmyopes	−0.048	0.008	−0.48	0.002
	Myopes	−0.088	0.008	−0.66	< 0.0001
LT					
	Nonmyopes	0.036	0.006	0.52	< 0.0001
	Myopes	0.063	0.005	0.66	< 0.0001
ASL					
	Nonmyopes	0.116	0.021	0.47	0.002
	Myopes	0.192	0.019	0.58	< 0.0001

All anterior segment parameters are in micrometers.

* Based on the estimate of fixed effects from the LMM analyses (regression slopes were significantly different between the refractive error groups for all parameters, *P* ≤ 0.007).

### Association Between Baseline Axial Length and Accommodation-Induced Changes in Axial Length

**Figure 6.**
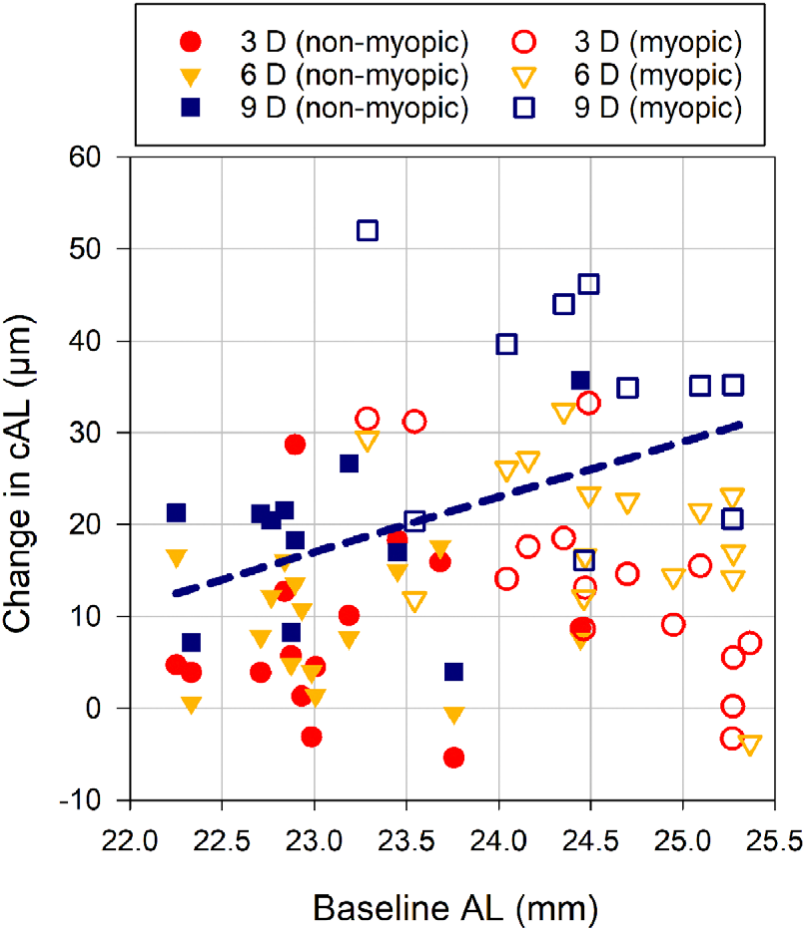
Scatterplot showing the association between the change in cAL at the 3 (*circles*), 6 (*triangles*), and 9 D (*squares*) accommodation stimuli and baseline AL (i.e., for the 0 D stimulus) for the nonmyopic (*filled symbols*) and myopic children (*open symbols*). The *dashed line* represents the linear regression fit as calculated from the estimate of fixed effects in the LMM analyses for the 9 D stimulus (Regression equation: Δ*cAL* = −121 + 6  × *AL*, where cAL is in micrometers and AL is in millimeters), with the Pearson correlation coefficient of *r* = 0.46 (*P* = 0.04).

A significant interaction was observed between the baseline AL and the change in cAL (*P* = 0.01), with a significant slope observed for the 9 D accommodation stimulus (*P* = 0.002), such that there was a 6 ± 2 µm (slope estimate ± SE of the estimate) increase in corrected axial elongation for every 1 mm increase in baseline AL (Fig. 6).

## Discussion

This is the first study to examine differences in the accommodation-induced ocular biometric changes between myopic and nonmyopic children. All axial biometric parameters, except CCT, changed significantly during accommodation, which confirms the results of our previous study examining nonmyopic children^18^ and prior studies of adults.^14^^–^^16^^,^^19^^,^^20^^,^^23^ The magnitude of the cAL changes and estimated AL measurement errors during accommodation for all participants were comparable to previous studies of children^18^ and adults.^16^ The myopic children exhibited significantly greater levels of axial elongation than the nonmyopic children at the 3, 6, and 9 D stimuli but displayed similar anterior segment changes during accommodation, which suggests that eyes with established myopia may be more susceptible to accommodation-induced axial elongation than nonmyopic eyes during childhood.

In young adults, Mallen et al.^15^ similarly observed significantly greater levels of axial elongation for myopes than emmetropes for a 6 D accommodation stimulus, although the magnitude of axial elongation at the same accommodation stimulus was substantially greater than in this experiment, likely due to their lack of correction of AL for the increased ocular refractive index during accommodation.^22^ Drexler et al.^14^ reported similar levels of AL elongation to the present study but conversely found that young adult emmetropes exhibited significantly greater axial elongation than myopes; however, the AL measurements of the myopic group were performed at an average accommodation stimulus of ∼1 D less than the emmetropic group. Read et al.^16^ was the most similar in experimental design to the present study and found no significant difference in the pattern of AL increases during accommodation between young adult myopes and emmetropes, with the level of AL elongation in both groups similar in magnitude to the nonmyopic children in the current study.

Although these AL changes are small and contribute ≤ 0.07 D to the change in refractive power of the eye associated with accommodation, the greater AL elongation in the myopic children in the present study may indicate an increased susceptibility of the myopic eye to elongate during childhood with increasing accommodation, a time when myopia is typically developing.^24^^–^^26^ Alternatively, this finding could reflect attenuation of accommodation-induced axial elongation with increasing age, as reported throughout adulthood.^27^ Previous studies have suggested that higher levels of myopia in adults^17^ and longer AL in nonmyopic children^18^ are associated with greater levels of accommodation-induced axial elongation, with the latter finding supported by the results of the present study.

The difference in the level of accommodation-induced AL changes between the refractive groups appears to be unrelated to the magnitude of the anterior eye changes during accommodation, as evidenced by the significantly steeper slopes for the associations between accommodation-induced axial elongation and the changes in ACD, LT, and ASL for the myopic children compared to the nonmyopic children. These findings suggest that both refractive error groups produced a similar accommodation response to the stimuli and that the greater accommodation-induced axial elongation in the myopic children is likely the result of other factors.

During accommodation, biomechanical forces associated with the anterior and inward displacement of the ciliary body^28^ are thought to produce generalized choroidal thinning^29^ and result in axial elongation.^30^ It is also possible that the autonomic innervation, responsible for driving the accommodation response,^31^ simultaneously stimulates contraction of the choroidal nonvascular smooth muscle (CNVSM)^32^ and produces choroidal thinning and axial elongation. More choroidal thinning during accommodation has been reported in myopes compared to emmetropes,^30^ which suggests that the myopic choroid may have a greater propensity to thin, possibly arising from differences in the distribution of autonomic innervation across the CNVSM, or the sensitivity of the CNVSM to autonomic input between nonmyopes and myopes.^30^

Alternatively, Drexler et al.^14^ and Mallen et al.^15^ proposed that the biomechanical forces during accommodation could reduce the equatorial diameter of the globe, and by volume redistribution, produce an AL increase. The myopic eye typically has a thinner than normal sclera,^33^^,^^34^ exaggerated at the posterior pole,^35^ potentially making it more susceptible to deformation^36^ under the influence of biomechanical forces linked to accommodation.^14^^,^^15^ Using the anterior sclera as a proxy for the posterior sclera, Woodman-Pieterse et al.^37^ demonstrated that the anterior sclera thinned during 3 and 6 D of accommodation in young adult myopes; however, emmetropes showed no significant changes. The greater axial elongation observed in the myopic children in the current study may reflect these differences in scleral biomechanical properties between myopic and nonmyopic eyes.

Although further research is required to ascertain the exact mechanisms underpinning accommodation-induced axial elongation, the results of this study support the hypothesis of a potential role of accommodation and near work in the development of childhood myopia, particularly relating to near work intensity and closer working distances. Compared to the amount of time spent engaged in near work activities, stronger associations between myopia prevalence and intense near work have been reported, examples being continuous reading without breaks^38^^–^^40^ and use of working distances under 30 cm during near tasks.^38^^,^^39^ In particular, the significantly increased level of axial elongation at the 6 and 9 D accommodation stimuli in this experiment (i.e., working distances of 17 and 11 cm, respectively) are consistent with the above hypothesis linking myopia with short working distances.

Recent studies of children in China^38^ and Australia^41^ have demonstrated that children regularly use short working distances for near work tasks during the school day, from under 10 cm to ∼25 cm, which encompasses the distances corresponding to the high accommodation stimuli (≥6 D) in this study. It is therefore possible that the axial elongation experienced by children during typical near work tasks may result in longer-term eye growth because of summation of accommodation-induced eye elongation, with the eyes of myopic children potentially being more susceptible. However, this study only examined biometric changes during a brief accommodation task (∼30–60 seconds), compared to the cumulative near work tasks of two to four hours per day on average reported in school children in Australia^41^^,^^42^ and Singapore.^43^ Furthermore, although our study findings may implicate accommodation-induced eye elongation in myopia development and progression, alternatively, they may simply reflect structural differences in the ocular tissues of myopic compared to nonmyopic eyes.

There are several limitations of this experiment. First, there was no simultaneous measurement of ocular biometry and accommodation response; however, only those participants who demonstrated a decrease and increase in ACD and LT, respectively, were included. Second, it is possible that the lack of proximity cues and the reduced field of view within the Badal optometer resulted in a reduced accommodation response^44^; however, this is likely to have resulted in an underestimation of the AL changes observed in this study. Third, the study sample size was small, particularly for the 9 D stimulus, which may have limited the ability to detect subtle differences in accommodation-induced changes between the refractive error groups. Finally, collecting data on young children can be challenging because of issues such as poor fixation or lack of task engagement; however, this was managed using various data collection strategies and maximizing task engagement through interesting fixation targets and repeating measurements when required.

Further research is required to understand the potential mechanisms underlying the accommodation-induced biometric changes in children, in addition to biometric changes during, and the recovery from, a prolonged accommodation task. Longitudinal observations of refractive error development, AL, and biometric changes during accommodation are also required before and after myopia development to investigate a potential causative link between accommodation, eye growth, and myopia development.

In conclusion, several ocular biometric parameters changed significantly during accommodation in children, which confirms previous findings in adults and nonmyopic children. Myopic children exhibited greater axial elongation during accommodation than nonmyopic children but displayed similar changes in all other biometric parameters. These findings may be the result of changes in the posterior structures of the eye associated with myopia development, such as the choroid or sclera. These results could support the hypothesis of a causative association between near work, accommodation, and myopia development; however, further studies are required to provide greater understanding of the link between accommodation-induced AL changes and refractive error development and eye growth.
